# Homozygous variant of MLC1 results in megalencephalic leukoencephalopathy with subcortical cysts

**DOI:** 10.1002/mgg3.2394

**Published:** 2024-02-09

**Authors:** Jian Zha, Yong Chen, Fangfang Cao, Yuxin Xu, Zuozhen Yang, Shu Wen, Mengmeng Liang, Huaping Wu, Jianmin Zhong

**Affiliations:** ^1^ Department of Neurology Jiangxi Provincial Children's Hospital Nanchang Jiangxi China; ^2^ Cipher Gene LLC Beijing China

**Keywords:** intellectual disorder, macrocephaly, megalencephalic leukoencephalopathy, *MLC1*

## Abstract

**Background:**

Megalencephalic leukoencephalopathy with subcortical cysts (MLC) is a rare, inherited disorder that causes epilepsy, intellectual disorders, and early onset macrocephaly. *MLC1* has been identified as a main pathogenic gene.

**Methods:**

Clinical data such as magnetic resonance imaging (MRI), routine blood tests, and physical examinations were collected from proband. Trio whole‐exome sequencing (WES) of the family was performed, and all variants with a minor allele frequency (<0.01) in the exon and canonical splicing sites were selected for further pathogenic evaluation. Candidate variants were validated using Sanger sequencing.

**Results:**

Here, we report a new homozygous variant identified in two children from the same family in the *MLC1* gene [NM_015166.4: c.838_843delinsATTTTA, (p.Ser280_Phe281delinsIleLeu)]. This variant is classified as variant of uncertain significance (VUS) according to the ACMG guidelines. Further experiments demonstrate that the newly identified variant causes a decrease of MLC1 protein levels when expressed in a heterologous expression system.

**Conclusion:**

Our case expands on this genetic variation and provides new evidence for the clinical diagnosis of *MLC1*‐related MLC.

## Graphical abstract

Here, we report a new and rare homozygous variant identified from two children from one family in MLC1 which was also investigated its role on protein by experiment.

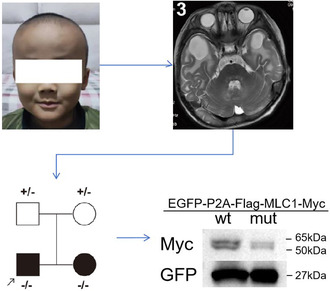

## 1 INTRODUCTION

Megalencephalic leukoencephalopathy with subcortical cysts (MLC) is a rare demyelinating disease of the central nervous system. Typical clinical presentation includes macrocephaly (an enlarged head) within the first year of life, delayed or normal intellectual development, and progressive deterioration of motor function over time. Individuals may also experience ataxia, seizures, spastic paraparesis, and extrapyramidal abnormalities. Magnetic resonance imaging (MRI) usually exhibits symmetric, diffuse, abnormally high signals in the bilateral cerebral white matter, with or without cystic lesions beneath the frontal and temporal cortices (Blattner et al., 2003; van der Knaap et al., 1995).

In 2001, *MLC1* was reported as the first causative gene for MLC (OMIM: 605908). *MLC1* is located on chromosome 22q13.33. Approximately 75% of individuals with MCL carry pathogenic variants of *MLC1* in an autosomal recessive heritage model (Leegwater et al., 2001).

In 2011, GlialCAM was identified as the second causative gene of MLC, and the disease phenotype associated with GlialCAM variants was named *MLC2*. Based on the different modes of inheritance, *MLC2* is further classified into two types: *MLC2A*, inherited in an autosomal recessive model; and *MLC2B* (MIM 613926), inherited in an autosomal dominant model. *MLC2* patients account for 20% of all MLC cases. *MLC2A* and *MLC2B* present with distinct clinical symptoms and prognoses. *MLC2A* is characterized by the progressive deterioration of motor function with age, similar to *MLC1*. However, *MLC2B*, also known as the remitting form, shows clinical and MRI improvements in some individuals after the age of one, and some may even achieve complete recovery (Hamilton et al., 2018; Lopez‐Hernandez, Ridder, et al., 2011; Lopez‐Hernandez, Sirisi, et al., 2011).

Here, we report two cases of classical MLC. Genetic testing reveals a homozygous variant of *MLC1* in both the proband and his elder sister. Experimental verification confirms that this new variant influences MLC1 protein expression. Our report contributes to the variant spectrum of *MLC1* related MLC and provides new evidence for its clinical diagnosis.

## 2 METHODS

### 2.1 Ethical compliance and clinical examination

This study was approved by the Institutional Ethics Board of Jiangxi Provincial Children's Hospital (no. JXSETYY‐YXKY‐20210053), and informed consent was obtained from the patient's parents. Routine blood tests, tandem mass spectrometry, plasma ammonia, lactate β‐Hydroxybutyric acid, electrolyte, blood glucose, electroencephalogram (EEGs), and magnetic resonance imaging (MRI) were performed, and all clinical information was collected and investigated. All the procedures were performed in accordance with the Declaration of Helsinki (2013 revision).

### 2.2 Genetic testing

Blood samples were collected from the patient and his family. Genomic DNAs was extracted from blood and purified by TIANamp Blood DNA Kit (DP348‐02, TIANGEN, China) following the manufacturer's guide. After purification, 1ug genomic DNA from each sample was first sheered into fragments, purified, then captured using the XGen Exome Research Panel (IDT, USA) following the manufacturer's guide, finally the libraries were sequenced on the NovaSeq 6000 Sequencing Platform. The raw data generated by the sequencer were converted to to fastq format data by bcl2fastq (https://github.com/savytskanatalia/bcl2fastq). After removing low‐quality reads and adaptors by TrimGalore (https://github.com/FelixKrueger/TrimGalore), the paired‐end clean reads were mapped to the human reference genome (GRCh38/hg38) with Burrows‐Wheeler Aligner (BWA) (Li & Durbin, 2009). Variant calling was performed using the Genome Analysis Toolkit (GATK) (Van der Auwera et al., 2013), and obtained variants were annotated using ANNOVAR (Wang et al., 2010). Variants located in exons or classical alternative splicing regions with a low frequency (<0.01) in public gnomAD database were obtained for further pathogenicity evaluation. The pathogenicity of the variants was classified according to the ACMG guidelines (Richards et al., 2015). Candidate pathogenic variants were confirmed by Sanger sequencing among the patient's family.

### 2.3 RNA extraction and *MLC1* cloning procedures

Total RNA was extracted from blood using the RNAiso Plus kit (108/9109, TaKaRa) and then reverse‐transcribed to cDNA for the construction of the expression vector as follows. Full‐length open reading frame sequences of the wild‐type and variant fragment of *MLC1* were amplified from the cDNA from the proband and his father respectively by *MLC1*‐GFP‐*BamH*I‐F (5′‐GCTTGGTACCGAGCTCGGATCCATGACCCAGGAGCCATTCAGAGAG‐3′) and *MLC1*‐GFP‐*EcoRI*‐R (5′‐GTGCTGGATATCTGCAGAATTCCTGGGCCATTTGCACCACGAC‐3′). After validation by Sanger sequencing, the wild‐type and variant fragments, pcDNA3.1‐EGFP‐P2A‐MYC vector (constructed from the pcDNA3.1 (v790‐20, addgene, MA, USA) as the plasmid backbone, reference sequence refers to the attachment) were digested with BamHI (1010S, Takara, Japan) and EcoRI (1040S, Takara, Japan) respectively following the manufacturer's guide. Then the wild‐type and variant fragments were ligated to pcDNA3.1‐EGFP‐P2A‐MYC vector respectively by T4 DNA ligase (2011A, Takara, Japan) following the manufacturer's guide. The vectors were transfected into the 293T cell line using Lipofectamine 2000 following the manufacturer's instructions. After 24 h, the cell was harvested for the following experiment.

### 2.4 qPCR

Total RNA was extracted from 293T cells using an RNA‐easy Isolation Reagent kit (R071; Vazyme Biotech, China). First‐strand cDNA was synthesized using (R212‐01, Vazyme Biotech). qPCR analysis was performed using SYBR Green Real‐Time PCR Master Mix (QPK‐201, Toyobo, Osaka, Japan) in a CFX Connect Real‐Time PCR System (Bio‐Rad, Hercules, CA, USA). Data were obtained via a standard curve analysis and normalized to an internal control gene (ACTB).

### 2.5 Western blot

Proteins were extracted from 293T cells using the RIPA lysis and extraction buffer (P0013B, Beyotime, Shanghai, China). Protein levels were measured using a bicinchoninic acid (BCA) assay kit (P0010; Beyotime, Shanghai, China). Total protein (~20 μg/lane) was subjected to SDS‐PAGE, before being transferred to a polyvinylidene difluoride membrane, blocked with 5% skim milk for 2 h, and incubated with mouse polyclonal anti‐GFP tag (1:4000, AE012, Abclonal, Wuhan, China) and anti‐GAPDH (1:1000, AF0006, beyotime, Shanghai, China) at 4°C overnight.

### 2.6 Statistical analyses

Data from independent experiments were presented as mean ± SEM. qPCR results were analyzed using a nonparametric test, and *p* < 0.05 were considered statistically significant. The significance between groups was calculated using GraphPad Prism 8 (GraphPad, USA).

## 3 RESULTS

### 3.1 Case presentation

A 4‐year‐and‐11‐month‐old boy was admitted to our hospital for having experienced seizures following a fall. The seizures were characterized by confusion, both eyes fixed gaze deviation to left, diminished responsiveness, pale lips, rigid limbs, and limb twitching. The episode lasted approximately 10 min, and the patient exhibited a poor mental state and excessive sleepiness afterward.

**FIGURE 1:**
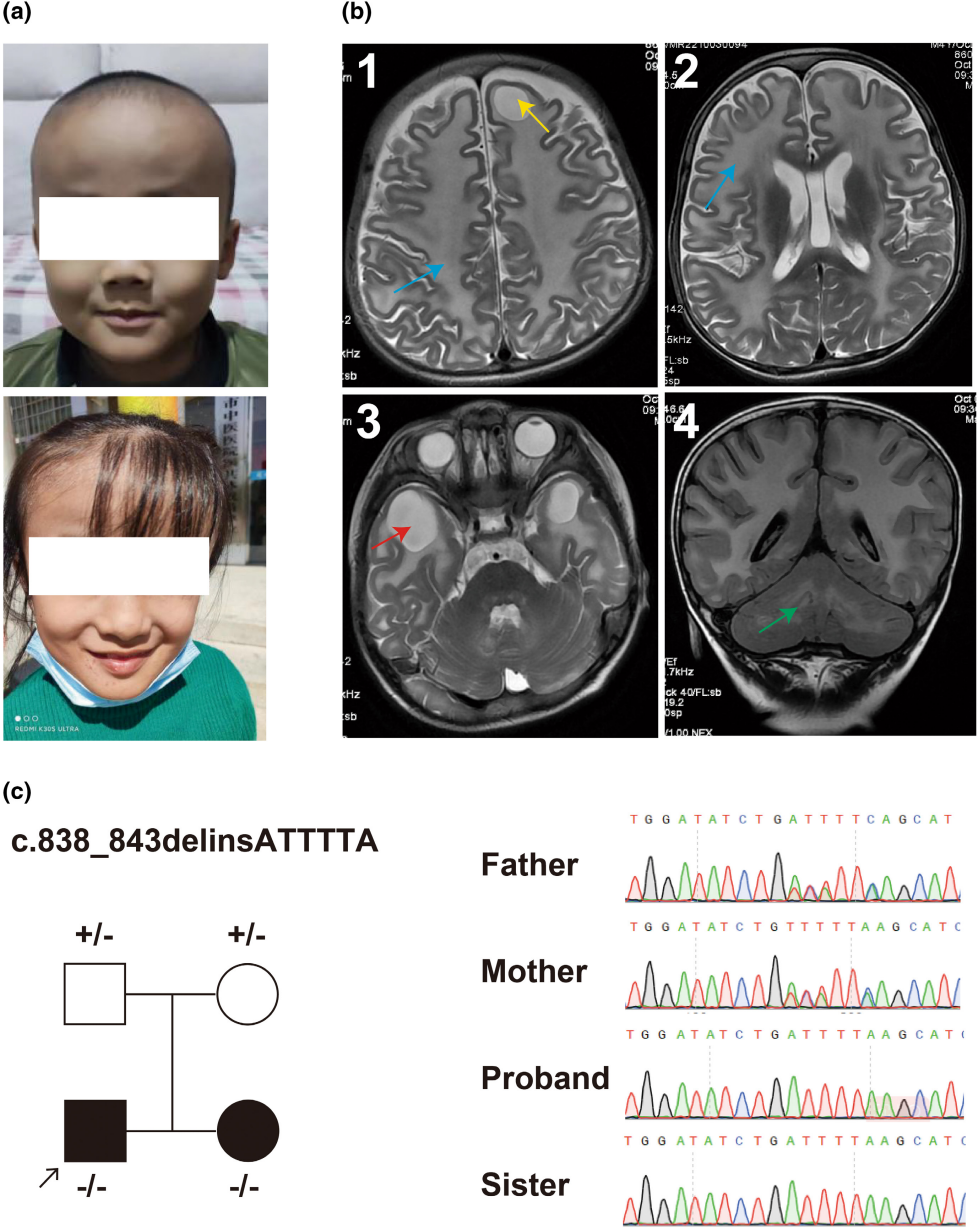
Clinical features of *MLC1* genetic variant. (a) Facial photographs of the proband and his elder sister. Both individuals exhibited macrocephalic abnormalities. (b) Brain MRI of the proband revealed diffuse swelling of the white matter, accompanied by cysts in the frontal and temporal lobes, as well as mild abnormal signals in the cerebellar white matter. 1: diffuse white matter swelling in the bilateral cerebral hemisphere (blue arrow), with high signal intensity on T2WI; accompanying frontal lobe cyst (yellow arrow); 2: diffuse white matter swelling in the bilateral cerebral hemisphere (blue arrow), with high signal intensity on T2WI; 3: temporal lobe cysts (red arrow); 4: mild abnormal signals in the cerebellar white matter (green arrow). (c) Pedigree chart, genotype, and variant validation by Sanger sequencing in the family.

At 1 year of age, he exhibited a large head circumference and started to walk around 2 years of age. He still had an unstable gait, quick walking with a tendency to lean forward, and poor jumping ability. His elder sister had a similar medical history, with a large head circumference (Figure 1a) and abnormal gait, diagnosed with “leukodystrophy.”

He exhibited slightly increased muscle tone, unsteady gait, Romberg sign, a positive finger‐nose test indicating ataxia, and a hyperactive knee reflex. No rashes or depigmented patches were observed. Cardiovascular and abdominal examinations, routine biochemical markers, tandem mass spectrometry, plasma ammonia, lactate, β‐hydroxybutyrate, electrolytes, and blood glucose showed no abnormalities.

Head MRI revealed the diffuse white matter swelling in the bilateral cerebral hemisphere accompanying frontal lobe cyst (Figure 1b(1,2)), temporal lobe cysts (Figure 1b(3)) and mild abnormal signals in the cerebellar white matter (Figure 1b(4)). Electroencephalography showed slow background activity. The developmental quotient (DQ) assessment indicated abnormalities (score: 44), with the most prominent impairment score (23.7) in gross motor skills.

Comprehensively considering the phenotype (head malformation, leukodystrophy, cerebellar ataxia, spasticity, epilepsy, etc.) and family history, he was highly suspected to have MLC. Blood samples were collected to perform exome sequencing for the identification of potential genetic variants.

### 3.2 Genetic testing

Trio whole‐exome sequencing (WES) was performed to screen for potential pathogenic genetic factors associated with neurodevelopmental disorders and epilepsy. A homozygous variant of *MLC1* [NM_015166.4: c.838_843delinsATTTTA (p.Ser280_Phe281delinsIleLeu)] was detected in both the proband and his older sister (Figure 1c). The variant was a 6 bp nucleotide deletion and another 6 bp nucleotide insertion, causing the substitution of the two SerPhe amino acids (280th and 281th nucleotides) to IleLeu. It was classified as variant of uncertain significance (VUS) for pathogenicity evidence: PM2_supporting (with a frequency of 0 in gnomAD), PM3_supporting (a homozygous variant detected in an autosomal recessive disorder), and PP1 (the variant was segregated in families). No other potentially pathogenic variants, including copy number variants (CNV), were detected.

### 3.3 Evaluation of the effects of the new variant on *MLC1* expression

**FIGURE 2:**
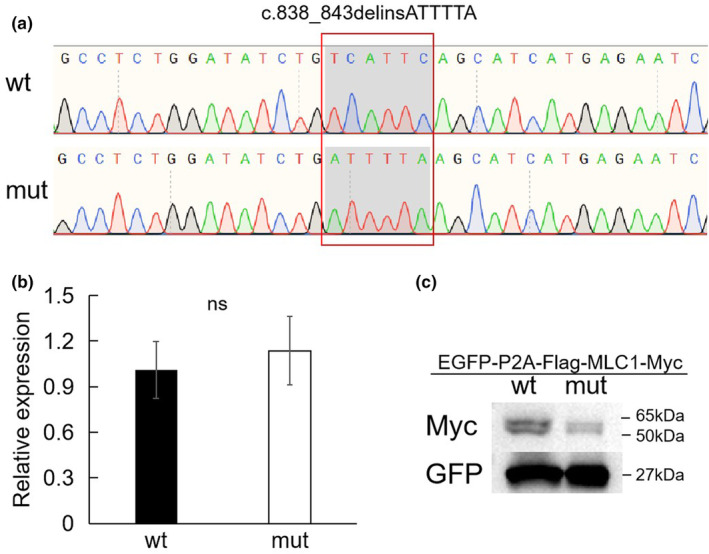
Evaluation the effect of the variant by experiments. (a) Sanger sequencing analysis to confirm MLC wild‐type and c.838_843delinsATTTTA variant cloning in the overexpression vector. (b) qPCR analysis for the quantification of wild‐type and variant (c.838_843delinsATTTTA) RNA expression. Expression levels were normalized to those of the ACTB control. ns, *p* > 0.05. Three technical replicates were performed for each treatment group. (c) Western blot analysis for the evaluation of MLC1 wild‐type and c.838_843delinsATTTTA variant protein expression.

To evaluate the effect of this variant, wild‐type and variant *MLC1* overexpression experiments were designed. The cloning of wild‐type and variant of *MLC1* in the vector were first confirmed using Sanger sequencing (Figure 2a). No statistically significant differences of RNA levels were observed between the wild‐type and variant by qPCR analysis (Figure 2b); however, a decreased level of the variant protein was observed when compared to the wild‐type one by western blot experiment (Figure 2c).

## 4 DISCUSSION

MLC is a rare autosomal recessive neurological disorder with a low incidence (approximately 1/100,000) and is characterized by progressive macrocephaly, white matter abnormalities, and subcortical cysts. Its main clinical features vary and it can develop into a serious syndrome including macrocephaly, ataxia, spasticity, seizures, mild to severe cognitive impairment, language developmental delays, optic atrophy, and nystagmus (Ashrafi et al., 2020; van der Knaap et al., 1993).

*MLC1* has been reported to be the first and main pathogenic gene in MLC. Currently, a total of 88 likely pathogenic or pathogenic variants have been collected in the ClinVar database (https://www.ncbi.nlm.nih.gov/clinvar/?term=MLC1%5Bgene%5D&redir=gene). Notably, only two deletion–insertion (delins) variants are reported (Choi et al., 2017; Leegwater et al., 2001; Tsujino et al., 2003), and both of them resulted in MLC1 loss of function.

Delins variants, also known as insertion–deletion (indel) variants, cause both the insertion and deletion of nucleotides. The effects of delins variants on proteins may be diverse and context dependent. If a delins variant disrupts the reading frame of a gene, it can result in a frameshift in the coding sequence, altering the subsequent amino acid sequence, and leading to a truncated or nonfunctional protein. It also causes the insertion or deletion of one or more amino acids in proteins that influences their structure, folding, stability, protein–protein interactions, enzymatic activity, or functional domains, leading to altered protein function. In our study, we discovered another delins variant that resulted in two amino acids substitutions. Around our variant, three other variants (all at 280th amino acid of MLC1) which linked to MLC are collected at ClinVar database: the c.838T>C (p.Ser280Pro) variant which classified as VUS (accession number:VCV001998382.1), the c.839C>A (p.Ser280Ter) variant which classified as Likely pathogenic (accession number:VCV000983677.3), and the c.839C>T (p.Ser280Leu) variant which classified as VUS (accession number:VCV000004713.1).

Previous studies have already described lower levels of expression of MLC1 pathological variants, either in patient derived cells (Petrini et al., 2013) or when expressed in heterologous expression systems (Duarri et al., 2008), mostly due to endoplasmic reticulum‐ and endo‐lysosomal‐associated degradation of the misfolded MLC1 variants (Duarri et al., 2008).

Analysis of brain tissue samples from a patient expressing a pathological variant of MLC1 confirmed that pathological mutations an affect MLC1 protein stability and reduce its expression in the brain (Lopez‐Hernandez, Ridder, et al., 2011; Lopez‐Hernandez, Sirisi, et al., 2011). Mutation proteins also exhibited trafficking defects and imparied localization at the plasma menbrane (Hwang et al., 2019; Petrini et al., 2013) causing alterations of the stable cell–cell communication and of the astrocyte‐mediated homeostatic control in the MLC brain (Estevez et al., 2018; Hwang et al., 2019; Lopez‐Hernandez, Ridder, et al., 2011; Lopez‐Hernandez, Sirisi, et al., 2011).

Currently, there is no effective treatment strategy for MLC, and almost all treatments focus on managing symptoms and providing supportive care such as physical therapy, occupational therapy, and antiepileptic medications for seizures (Ashrafi et al., 2020). Considering the treatment status, a precise diagnosis and genetic counseling are important. Experiments to validate the effect of the MLC1 protein are crucial for strengthening the pathogenicity evidence of the variant, especially in the case of the many variants are still classified as VUS in the ClinVar database.

## SUMMARY

In this study, we reported two MLC Individuals carrying the same homozygous variant of *MLC1*. Our experiments demonstrated the amino acides substitutions found in the newly described variant affect MLC1 protein stability. Our cases expand the variant spectrum of *MLC1* related MLC, underlying the importance of genetic testing and counseling in clinics.

## AUTHOR CONTRIBUTIONS

JZ and YC: conceived the study and wrote the first draft of the manuscript. HPW and JMZ: helped critically revise the manuscript for important intellectual content and were the mentors who designed and guided the research study. ZZY, SW, and MML: carried out the variant analyses. FFC and YXX: oversaw patient care and collected the clinical data. All authors contributed to the article and approved the submitted version.

## CONFLICT OF INTEREST STATEMENT

All authors declare that there are no conflicts of interest.

## ETHICS STATEMENT

The study was approved by the Human Ethics Committees of Jiangxi Provincial Children’s Hospital and written informed consent was obtained from the legal guardians of the patient of any potentially identifiable images or data included in this article.

## Data Availability

The variant has been submitted to the ClinVar database (accession number: SUB13604828). The data that support the findings of this study are openly available in ClinVar at https://www.ncbi.nlm.nih.gov/clinvar/, reference number SUB13604828.
